# P-244. Blood Glucose Outcomes of Anti-retroviral Therapy Naïve Ugandan people with HIV with Pre-diabetes Mellitus Initiated on Dolutegravir for 48 weeks

**DOI:** 10.1093/ofid/ofaf695.466

**Published:** 2026-01-11

**Authors:** Frank Mulindwa, Barbara Castelnuovo, Jean-Marc Schwarz

**Affiliations:** United Health Services, Wilson Hospital, Johnson City, NY; Makerere University Infectious Diseases Institute, Kampala, Kampala, Uganda; University of California San Francisco, San Francisco, California

## Abstract

**Background:**

There have been case reports and series of persons with HIV (PWH) developing accelerated hyperglycemia on starting integrase inhibitors with some countries like Uganda adopting intensified blood glucose monitoring in PWH stratified as being at risk. We sought to determine if PWH with pre-diabetes on starting dolutegravir/ lamivudine and Tenofovir Disoproxil Fumarate had worse blood glucose outcomes compared to those with normal blood glucose at baseline.

**Table 1:**
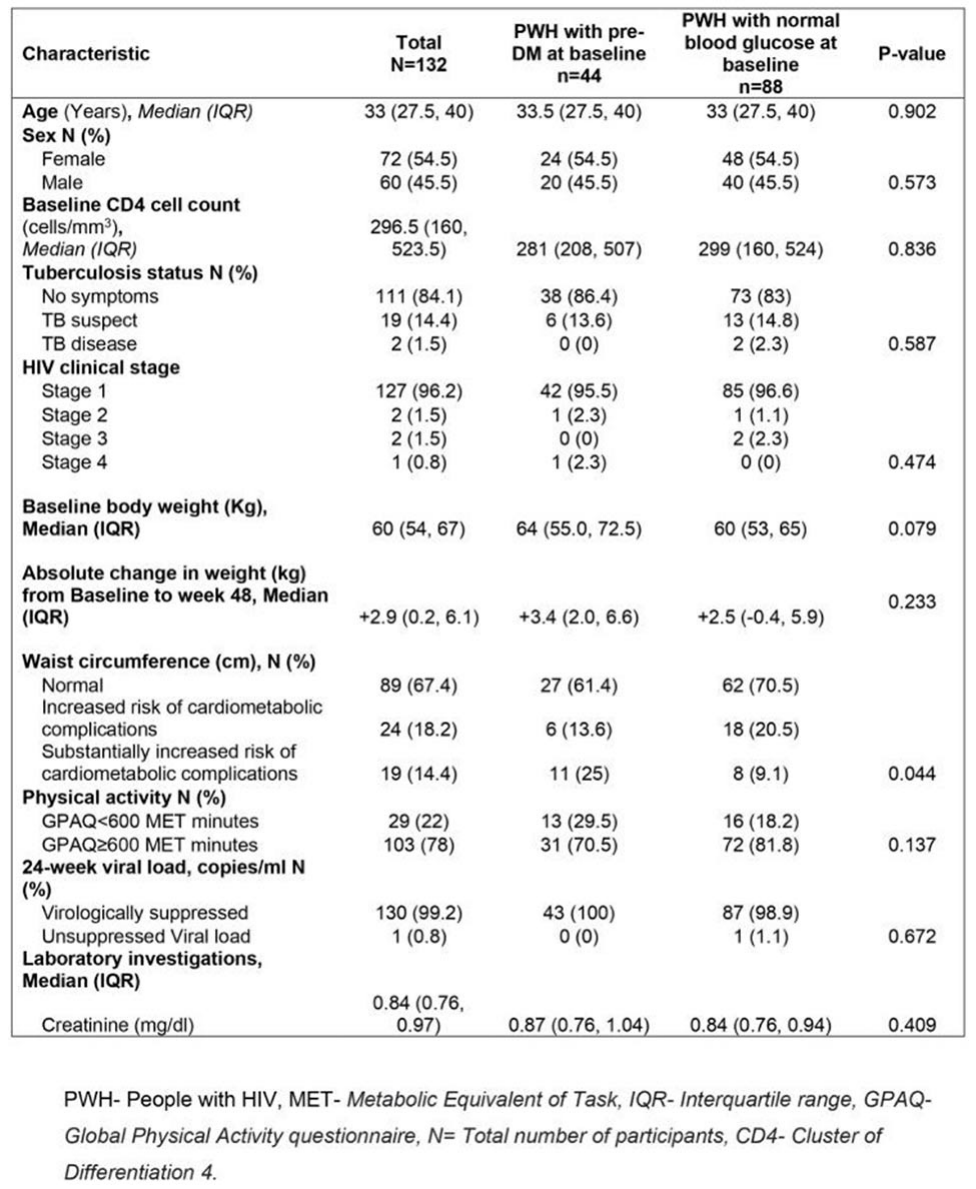
Baseline clinical and demographic characteristics compared between participants with pre-diabetes mellitus and those with normal blood glucose at baseline

**Table 2:**
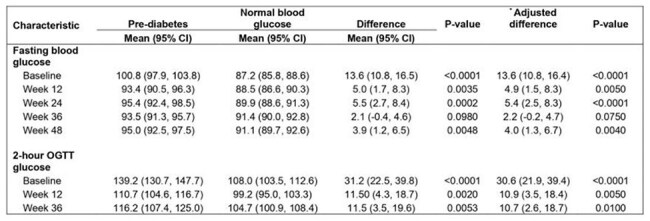
Differences in mean blood glucose at different time points compared between participants with pre-diabetes mellitus and those with normal blood glucose at baseline

**Methods:**

In this matched cohort study, we compared 44 PWH with pre-DM (pre-DM group) and 88 PWH with normal blood glucose (normal blood glucose group) at baseline. The primary outcome was change in mean fasting blood glucose (FBG) from baseline to week 48 and 2-hour blood glucose (2hBG) from baseline to week 36 compared between the two groups.

**Table 3:**
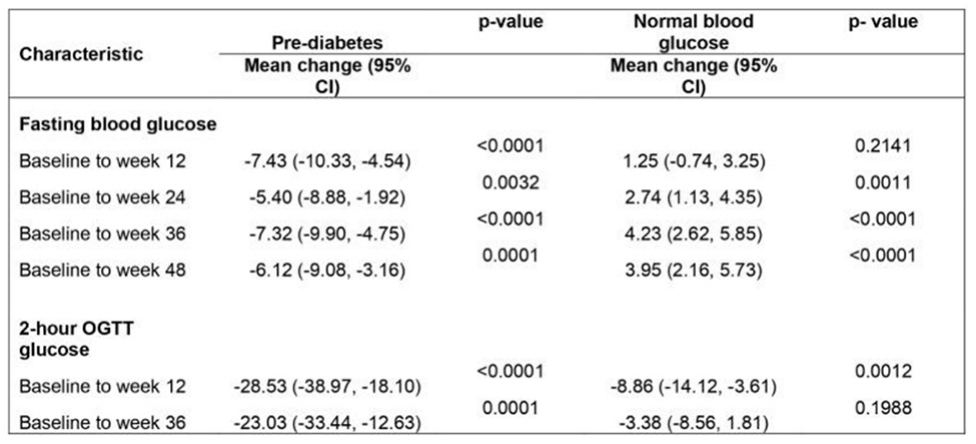
Mean blood glucose from baseline to end of follow up for participants with pre-diabetes mellitus and those with normal blood glucose at baseline

**Results:**

FBG was higher in the pre-DM group at all time points (Adjusted difference in FBG between the pre-DM group and normal blood glucose group at 12 weeks: 4.9 mg/dl, 95%CI (1.5, 8.3), p= 0.005, 24 weeks: 5.4 mg/dl, 95%CI (2.5, 8.3), p= < 0.0001, 36 weeks: 2.2 mg/dl, 95%CI (-0.2, 4.7), p= 0.04 and 48 weeks: 4.0 mg/dl, 95%CI (1.3, 6.7), p= 0.004). Similarly, 2-hBG was higher in the pre-DM group at 12 weeks (Adjusted difference in 2hBG between pre-DM group and normal blood glucose group: 10.9 mg/dl, 95%CI (22, 39.9), p= 0.005) and 36 weeks: 10.7 mg/dl, 95%CI (2.6, 18.7), p= 0.01). (Table 2).

Despite the pre-DM group having higher mean blood glucose at all time points, this group as well had improvement in FBG at 48 weeks of follow up (adjusted mean difference in FBG at 48 weeks from baseline:-6.12 mg/dl, 95%CI (-9.08, -3.16), p= 0.0001) and 2hBG at 48 weeks (adjusted mean difference in 2hBG at 48 weeks from baseline (a2hBG: -23.0 mg/dl, 95%CI (-33.4, -12.6), p= 0.0001). Table 3

**Conclusion:**

We demonstrated that this cohort of ART naïve PWH with pre-diabetes at enrollment have consistent improvement in both fasting blood glucose and glucose tolerance over 48 weeks on TDF/3TC/DTG just like their counterparts with normal blood glucose at baseline. Preferential intensified blood glucose monitoring of these patients in the first 48 weeks may be unnecessary.

**Disclosures:**

All Authors: No reported disclosures

